# Site‐specific responses of foliar fungal microbiomes to nutrient addition and herbivory at different spatial scales

**DOI:** 10.1002/ece3.5711

**Published:** 2019-10-19

**Authors:** Candice Y. Lumibao, Elizabeth T. Borer, Bradford Condon, Linda Kinkel, Georgiana May, Eric W. Seabloom

**Affiliations:** ^1^ Department of Ecology, Evolution and Behavior University of Minnesota St. Paul Minnesota; ^2^ Department of Plant Pathology University of Minnesota St. Paul Minnesota

**Keywords:** *Andropogon gerardii*, nitrogen, phosphorus, potassium, Nutrient Network, phylogenetic diversity, plant fungal endophytes, spatial variation

## Abstract

The plant microbiome can affect host function in many ways and characterizing the ecological factors that shape endophytic (microbes living inside host plant tissues) community diversity is a key step in understanding the impacts of environmental change on these communities. Phylogenetic relatedness among members of a community offers a way of quantifying phylogenetic diversity of a community and can provide insight into the ecological factors that shape endophyte microbiomes. We examined the effects of experimental nutrient addition and herbivory exclusion on the phylogenetic diversity of foliar fungal endophyte communities of the grass species *Andropogon gerardii* at four sites in the Great Plains of the central USA. Using amplicon sequencing, we characterized the effects of fertilization and herbivory on fungal community phylogenetic diversity at spatial scales that spanned within‐host to between sites across the Great Plains. Despite increasing fungal diversity and richness, at larger spatial scales, fungal microbiomes were composed of taxa showing random phylogenetic associations. Phylogenetic diversity did not differ systematically when summed across increasing spatial scales from a few meters within plots to hundreds of kilometers among sites. We observed substantial shifts in composition across sites, demonstrating distinct but similarly diverse fungal communities were maintained within sites across the region. In contrast, at the scale of within leaves, fungal communities tended to be comprised of closely related taxa regardless of the environment, but there were no shifts in phylogenetic composition among communities. We also found that nutrient addition (fertilization) and herbivory have varying effects at different sites. These results suggest that the direction and magnitude of the outcomes of environmental modifications likely depend on the spatial scale considered, and can also be constrained by regional site differences in microbial diversity and composition.

## Graphical abstract

The plant microbiome can affect host function in many ways yet our understanding of the ecological and environmental factors that shape plant microbiomes is limited, especially within the evolutionary context of phylogenetic relatedness among members of a community. We characterized the responses of foliar fungal endophytic microbiomes of *Andropogon gerardii* to nutrient addition and herbivory at different spatial scales in a distributed, regional‐scale experiment. Despite increasing fungal diversity and richness, phylogenetic diversity of fungal microbiomes did not differ systematically when summed across increasing spatial scales although nutrient addition and herbivory have varying effects at different sites, suggesting that that the direction and magnitude of the outcomes of environmental modifications likely depend on the spatial scale considered and can also be constrained by regional site differences in microbial diversity and composition.

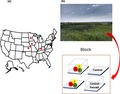

## 1 INTRODUCTION

The microbiome, the community of microbial species inhabiting an individual host, is composed of a diverse community of potentially interacting microorganisms (Vorholt, 2012). Organisms within plant microbiomes can play many key functional roles for hosts, including conferring benefits such as stress tolerance, nutrient acquisition, (Arnold & Lewis, 2005; Rodriguez et al., 2008) as well as facilitation and antagonism of pathogens (Busby, Ridout, & Newcombe, 2016; Christian, Herre, Mejia, & Clay, 2017). Like free‐living organisms, the capacity of the host‐associated microbiomes to confer benefits for hosts especially under environmental perturbations depends on both the identity and the diversity of the microbes comprising that community (Luo et al., 2016; Tian, Cao, & Zhang, 2015). Thus, it is important to characterize the impacts of environmental changes on plant microbiomes, such as endophytic fungal communities (i.e., those that live inside plant tissues).

Environmental changes in abiotic conditions such as host nutrient supply, and in biotic factors (e.g., herbivory or competition) across the landscape can influence the composition and diversity of microbial assemblages (Giauque & Hawkes, 2013; Kerekes et al., 2013; Lumibao et al., 2018; Pancher et al., 2012). Nutrient addition can alter host plant resources available to microbial symbionts, which can alter abundances of specific fungal taxa associated with the plant host. For instance, specific functional groups such as mycorrhizal fungi in plants and soils have been shown to decrease in relative abundance compared to other groups with nitrogen addition (Leff et al., 2015; Liu et al., 2015; Treseder, 2008; Wessén, Nyberg, Jansson, & Hallin, 2010), while fungal genera with known pathogenic traits increased in abundance (Paungfoo‐Lonhienne et al., 2015). Similarly, herbivory can alter microbial community composition within hosts via changes to host plant tissue chemistry, potential for increased colonization by serving as vectors, and regulating host plant immune systems (Cosme et al., 2016; González et al., 2018). While these studies provided insights into factors shaping patterns of plant‐associated microbial assemblages, our knowledge on the processes and mechanisms of response in microbial communities to anthropogenic or environmental changes remains limited, particularly within an evolutionary or phylogenetic context.

Phylogenetically related fungi may share similar ecological roles, and these phylogenetic relationships can offer potentially new insights into endophytic microbial community responses to environmental changes. In general, specific phylogenetic patterns may arise from both within and among communities depending on evolutionary lineages of taxa comprising a community (Mouquet et al., 2012; Webb, Ackerly, McPeek, & Donoghue, 2002). For example, if the taxa comprising a community share ecological traits or niches due to evolutionary history, they will also be more closely related than expected by chance (phylogenetically clustered community). On the other hand, if taxa within communities have little niche overlap due to a history of competition and resulting limiting similarity, the taxa in a community are expected to be more distantly related than expected by chance (phylogenetically over‐dispersed; Cavender‐Bares, Kozak, Fine, & Kembel, 2009; Webb et al., 2002; but see Mayfield & Levine, 2010). Evidence is accumulating that phylogenetic relationships among co‐occurring taxa can shape microbial responses to environmental change (e.g., Amend et al., 2016; Evans & Wallenstein, 2014; Treseder, Kivlin, & Hawkes, 2011). Studies also have found that environmental change may alter phylogenetic diversity; for example, under climatic changes, microbial communities tend to be dominated by taxa within a few clades (e.g., Barnard, Osborne, & Firestone, 2013; Placella, Brodie, & Firestone, 2012).

If species with shared ecological attributes and ancestry have similar responses to environmental pressures (Martiny, Treseder, & Pusch, 2013), we can expect that changes in the local environment such as nutrient addition and herbivory may influence phylogenetic diversity (measured as phylogenetic relatedness or similarity patterns) and composition of fungal assemblages. However, at different spatial scales ranging from few centimeters within hosts and few meters within plots to hundreds of kilometers among sites, the impact of these environmental changes on phylogenetic diversity patterns of communities might vary in strength depending on the relative importance of other processes such as competition, dispersal limitation or environmental filtering (Nekola & White, 1999; Webb et al., 2002). Thus, different phylogenetic patterns in response to environmental changes may be observed at different spatial scales. For instance, at smaller spatial scale, such as within‐host, local competition may be the predominant process, leading to selection of fungal taxa across evolutionary clades, that is, fungal taxa with limited ecological similarities or similarities in traits. Hence, a phylogenetically over‐dispersed community might be observed at small spatial scale (although the opposite pattern can also arise). On the other hand, at larger scale such as regional or site level, environmental filtering which can be further imposed by environmental perturbations, can lead to selection of closely related taxa with similar environmental tolerance, thus, resulting in phylogenetically clustered community.

Local fungal assemblages may be derived from regional fungal pools that are evolutionarily unique compared to other regions, as fungi can be dispersal‐limited (David, Seabloom, & May, 2016; Mouquet et al., 2012). Thus, we can also expect that regardless of environmental differences, phylogenetic relatedness of co‐occurring members of communities will decline with increasing spatial scale (Morlon et al., 2011). The decline in phylogenetic diversity can be accompanied by phylogenetic turnover (or shifts in composition, similar to phylogenetic beta‐diversity) among communities. Alternatively, if the regional pools of fungi harbor distinct but similarly diverse lineages of fungi, local communities may be composed of co‐occurring members with random phylogenetic associations, that is, drawn at random from diverse fungal pool. Thus, we might expect increasing levels of phylogenetic diversity at increasing spatial scales.

Characterizing phylogenetic diversity patterns in response to environmental differences across spatial scales can provide a deeper understanding of the key factors influencing assembly of fungal endophytic communities and provides a potentially new direction linking ecological and evolutionary processes. Here, we experimentally manipulated nutrient supply (fertilization) and herbivory, two key environmental factors that affect grassland ecosystems and replicated our experiment at four sites across the Midwestern United States. We assessed whether: (a) phylogenetic diversity of foliar fungal endophytic microbiomes would increase/decrease across spatial scales, that is, from within‐host to site‐level scale; and (b) the magnitude and direction of the effects of fertilization and herbivory on the phylogenetic diversity and composition of fungal microbiomes vary across different spatial scales. We expect that at site level, environmental filtering due to similar environmental tolerances of closely related fungal taxa will result in phylogenetically similar communities across different environmental modifications, although this might depend on the composition of regional pools of fungi at each site.

## 2 METHODS

### 2.1 Sample collection and site

**Figure 1:**
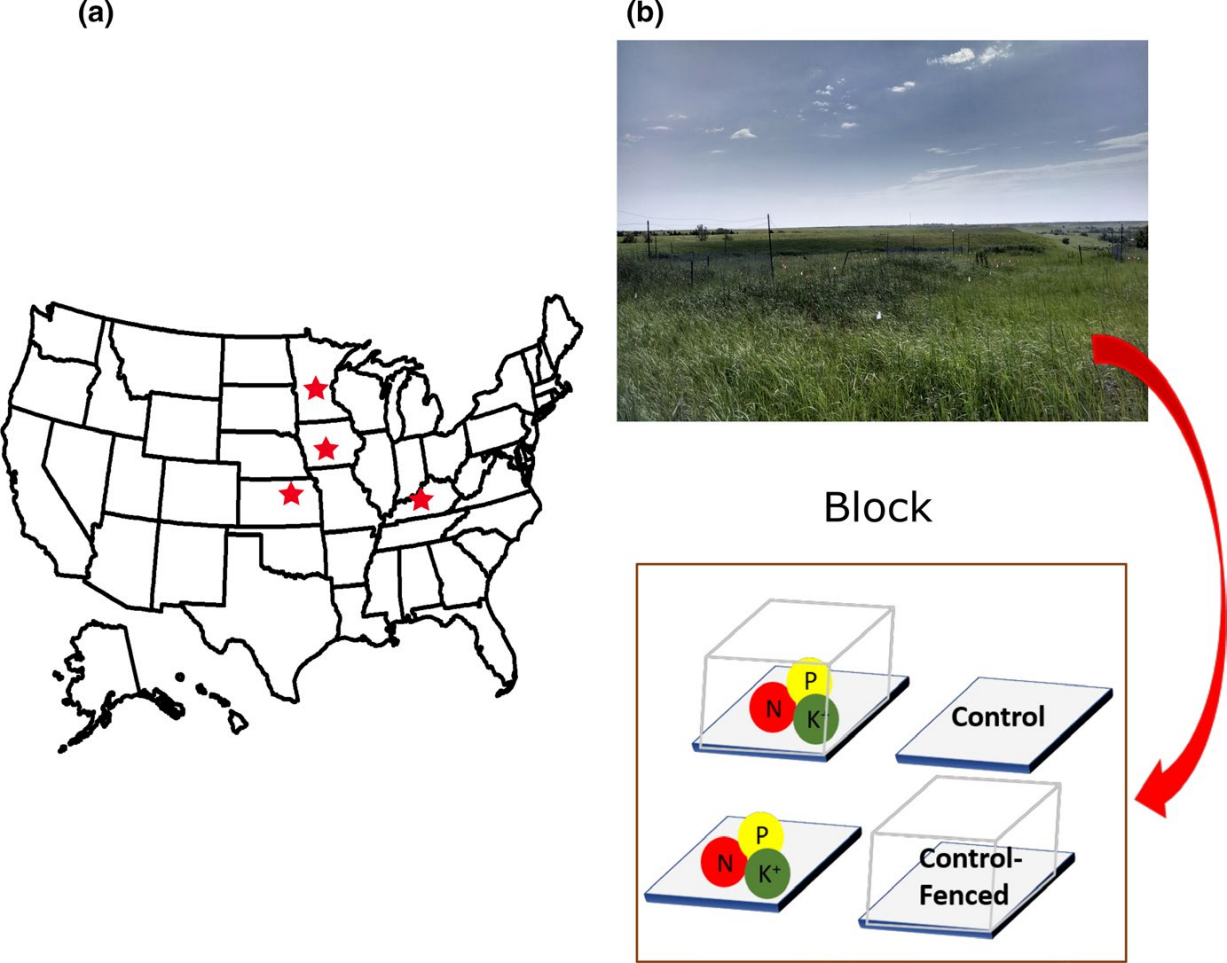
(a) Map of our regional sites and (b) the Nutrient Network (NutNet) experimental set‐up

Our focal host, *A. gerardii*, is a common perennial grass species native to the Midwest region of the USA. The oldest leaf from each plant was collected from experimental plots at four sites of the Nutrient Network (NutNet; http://nutnet.org), a global network of nutrient addition and herbivore exclosure experiments (Borer, Grace, Harpole, MacDougall, & Seabloom, 2017). The sites were located in Minnesota, Kentucky, Kansas, and Iowa (Figure 1a). NutNet field plots (1 × 1 m) were established in 2007 in Minnesota, Kentucky and Kansas and in 2008 in Iowa. We selected *A. gerardii* as our focal species as it is widespread and the only species present across all four sites. The four sites span a range of mean annual temperature (6.3–13.6°C) and precipitation (750–1,282 mm/year).

Plots consist of a factorial combination of nutrient fertilization and fences that exclude only large vertebrate herbivores (Figure 1b). Nutrients (nitrogen, phosphorous, potassium) were added as 10 g/m^2^/year in addition to a one‐time 100 g/m^2^ treatment with a micronutrient mix (6% Ca, 3% Mg, 12% S, 0.1% B, 0.1% Cu, 17% Fe, 2.5% Mn, 0.05% Mo, and 1% Zn) at the start of the experiment. The four treatment combinations—fertilization (NPK), fertilization with herbivore exclusion via fencing (NPK_H‐), herbivore exclusion without fertilization (C_H‐) and control (C)—were replicated in three blocks at each site. Herbivore exclusion treatments via fencing only exclude large herbivores, which were consistent within and across all sites (Borer et al., 2017). This allows us to examine the effect of modifying the foodweb in the same magnitude; in contrast to other smaller herbivores that were access the plots in ambient rate. Thus, this design allows us to isolate and quantify the impacts of large mammalian herbivores, a group that has been shown to have substantially reduced grazing in the presence of certain fungal endophytes, restructures the fungal endophytes within grass hosts within and among sites. In August 2014, at the time of peak biomass, leaf samples were collected from four different plants (one leaf per plant) in each of the block (one from each of the four treatment plots), except for Iowa where *Andropogon* was less common, so samples were collected from two blocks at this site. Thus, the study comprises 176 host individuals spanning sites and treatment plots.

Leaves were stored at 4°C in the field and returned to the laboratory where they were surface‐sterilized within 24 hr following collection. Surface sterilization was carried out in sequential manner for 1 min each with water, 75% ethanol, 0.4125% sodium hypochlorite (bleach solution), 75% ethanol and sterile distilled water for 1 min with each solution and stored at −80°C until DNA extraction.

### 2.2 Sequence analyses

Total genomic DNA was extracted from each leaf sample and then used to generate amplicons at the fungal barcode internal transcribed spacer 1 (ITS1) ribosomal DNA region. Leaves were ground in liquid nitrogen using mortar and pestle and total genomic DNA was extracted using the Qiagen Plant Mini Extraction Kit (Qiagen N.V.). Genomic DNA was standardized to 20 ng/μl and fungal genomic libraries were made by amplifying the ITS region as described in Nguyen, Smith, Peay, and Kennedy (2015). Briefly, each sample was barcoded or tagged with unique 7‐base pair sequences (Nguyen et al., 2015; Smith & Peay, 2014) and the ITS region was amplified with the standard primers ITS1f (5′‐AATGATACGGCACCACCGAGATCTACAC‐GG‐CTTGGTCATTTAGAGGAAGTAA‐3′) and ITS2 (5′‐CAAGCAGAAGACGGCATACGAGAT‐barcode‐CG‐GCTGCGTTCTTCATCGATGC‐3′). The ITS2 primer includes an Illumina Nextera adaptor, linker sequence, and a barcode. Polymerase chain reaction (PCR) was done in triplicate using Roche FastStart High Fidelity Taq (Roche) with annealing temperatures at 51, 53, and 55°C to amplify a wide range of fungal taxa and reduce amplification bias for short ITS sequences. PCR conditions were as follows: Initial denaturation 94°C 10 min, 30 cycles of 94°C 30 s, 51–55°C 15 s and 72°C for 30 s; final elongation at 72°C for 8 min. PCRs that failed the first time were redone to potentially account for technical errors during the PCR. Two negative controls (distilled water) were included in every PCR reaction. Amplicons from the triplicate PCRs were pooled for each sample, then purified using the QIAQuick Purification Kit (Qiagen N.V.) and quantified using the Quant‐iT^®^ dsDNA HS Assay kit in Qubit Flourometer (Thermo Fisher). Equal amounts of these purified libraries (25 ng) were pooled and sequenced in Illumina MiSeq at the University of Minnesota Genomics Center (UMGC).

### 2.3 Bioinformatics analyses and fungal taxa identification

Fungal community profiling was done by Operational Taxonomic Unit (OTU) clustering and taxa assignment. Sequence data from all the MiSeq runs were first combined so all samples can be analyzed simultaneously using the metagenomic pipeline adapted from Nguyen et al. (2015). Briefly, sequences were trimmed by removing adapter and distal priming sites using cutadapt v1.7.1 (Martin, 2011) with low‐quality read ends trimmed at 20 bp cutoff prior to adapter removal, followed by further removal of untrimmed low‐quality regions using Trimmomatic v 0.32 (Bolger, Lohse, & Usadel, 2014). Further filtering was conducted by removing short sequences, homopolymers up to 9 base pairs and sequences containing ambiguous bases in mothur v.1.34.4 (Schloss et al., 2009). The cleaned‐up sequences were then dereplicated, and clustered into OTUs using a double‐clustering approach (chain picking) adapted from Nguyen et al. (2015). OTUs were first clustered at a 97% cutoff, with chimera sequences removed as implemented in the program USEARCH (Edgar, 2013), followed by additional reclustering using uclust implemented in Qiime v1 (Caporaso et al., 2010), with the same 97% cutoff. Singleton OTUs (OTU with sequence count = 1) were removed from the pool. The resulting OTUs were then used in picking a representative sequence for each OTU for taxonomy assignment.

Operational Taxonomic Units were assigned taxonomy using BLAST (Basic Local Alignment Search Tool) alignment against the UNITE fungal database v 7.2 with BLAST v 2.2.28+ (Camacho et al., 2009). We set a threshold of 80% hit length alignment and of those alignment, 80% identity for inclusion (annotations below 80% on both metrics were excluded). Sequences from negative controls were pooled together, and for any OTU present in negative control, the resulting sequence read counts were subtracted from the sequence counts of that particular OTU in each of our samples. We recovered 748 sequence reads from the pooled negative controls, representing a fraction of total number of reads (<0.0001) clustered into 27 OTUs. Most of the OTUs were rare with median abundance of 4.5, except for one OTU classified *Phoma calidophila* comprising 72% of the total negative control reads. These are likely to be from technical noise from the sequencing, rather than anything biological, or contaminants in the water (though we do not discount that possibility).

For phylogenetic analyses, all OTUs matching the same taxa were collapsed into consensus taxa (following the software ghost‐tree method described below). Sequence counts for that particular taxon were aggregated, and the sum of combined counts was used as the abundances in the species abundance matrices for analyses. As the phylogenetic tree generated (described below) requires assignment to specific taxa, unassigned OTUs below the BLAST cutoff and those with no hits against UNITE database were also excluded from the analyses as we are interested in OTUs that we can specifically assign to a particular fungal taxon for reconstructing phylogenetic relationships across taxa.

### 2.4 Data analysis

We assessed the effects of fertilization and herbivore exclusion on the phylogenetic diversity of foliar endophytic fungal communities by estimating (a) within‐community phylogenetic diversity (similar to alpha diversity) across regions; and (b) at local scale, that is, within each regional site; and (c) shifts in phylogenetic composition among fungal communities—equivalent to phylogenetic beta‐diversity—among our four regional sites. In order to give more context to these results, we also calculated the nonphylogenetic diversity, for example, fungal richness, and compared patterns between phylogenetic and nonphylogenetic diversity. For the latter, we used only the OTUs that were included in the hybrid phylogenetic tree generated by ghost tree (see below). We tested for potential biases in using ghost‐tree subset data compared to the full dataset by testing for differences using nonphylogenetic metric of diversity (Shannon Diversity) and compositional distance (Bray–Curtis; see Section 2.5). All variables were transformed where necessary. Prior to all analyses, data were rarefied to 900 sequence counts using rrarefy function in vegan v2.3 (Oksanen et al., 2013). All analyses were done in R v3.5 (R Development Core Team, 2016).

### 2.5 Testing for biases in the OTU dataset

Of the total 2,772 OTUs delineated, about 57% (*n* = 1,577) were included in the phylogenetic analyses, representing 841 fungal taxa (i.e., included in the hybrid phylogenetic tree generated from ghost tree). We acknowledged that the exclusion of unidentified taxa poses some potential biases and caveats that can limit or influence our results, which we described in succeeding section. The number of fungal taxa that were included in the tree did not differ across site and treatments. As these represent only a subset of OTUs that were included in the phylogenetic analyses, we examined for potential biases this might introduce by analyzing the full dataset, that is, all OTUs prior to blast assignment, then comparing patterns between the full and subset dataset and testing for correlation using simple linear regression. Comparison of patterns between the full and subset OTU (i.e., those included in the phylogenetic analyses) dataset revealed significant correlations as results were qualitatively similar. Both Shannon diversity and OTU richness exhibited similar increasing patterns across spatial scales and were significantly correlated between the subset and full dataset at each spatial scale, that is, from leaf to site level (Appendix S1, Figures S1 and S2). In addition, PERMANOVA analysis also revealed similar stronger clustering by site than treatment using either the full or the subset dataset (Table S2). Thus, we conclude that no potential biases were introduced when using the full versus subset dataset.

### 2.6 Phylogenetic tree

Although the ITS1 region of rDNA locus in fungi typically allows identification of taxa, the high rate of mutation, especially due to insertion and deletions (indels), makes it challenging to reliably infer phylogenetic relationships among taxa. Thus, for fungal phylogeny, we used the phylogenetic tree generated using sequences from two regions, ITS and 18S SSU (small subunit) rRNA, using the open‐source bioinformatics tool ghost tree developed by Fouquier et al. (2016). Ghost tree is a pipeline for creating a hybrid phylogenetic tree (called a “ghost tree”) that integrates sequences from the two abovementioned regions. It uses the same principle in creating phylogenetic trees as in other studies that combine multiple genetic loci to reconstruct phylogenetic relationships (Fouquier et al., 2016). We used prebuilt phylogenetic tree that was built using UNITE v.7 and SILVA v132 SSU (Fouquier et al., 2016). See Appendix S1 for further details.

While the use of SSU and ITS regions in building the hybrid phylogenetic tree can potentially infer phylogenetic relationships among taxa, there are some limitations to the ghost‐tree approach. For instance, the pooling of OTUs and further reclustering by taxon label (genus level) mask variations among closely related taxa. Unassigned OTUs (below the 80% cutoff) were also excluded, which can also differ across sites and treatments, thus, might not reflect the entire fungal communities and potential patterns might be missed. These might pose limitations to our inferences, for example, if evolution of traits of interest is occurring at the species or population level, taxonomic resolution can be lost. Hence, we interpret and discuss our findings in the light of these caveats. We focused on the broad‐scale diversity patterns among and within communities, by assessing fungal assemblages in both phylogenetic and nonphylogenetic context. We also examined biases in our dataset by analyzing and comparing dataset that includes all OTUs and a subset dataset that includes only the OTUs incorporated in the phylogenetic tree. Finally, we do not seek to relate these to specific function or functional diversity, but rather infer phylogenetic relatedness among co‐occurring taxa within a community.

### 2.7 Phylogenetic diversity metric

While there are many metrics for assessing phylogenetic diversity within communities, we used the mean phylogenetic distance (MPD; Webb, 2000) weighted by abundance. MPD is less sensitive to the number of species in a sample. It provides a measure of phylogenetic diversity by taking the mean phylogenetic distance between all pairs of individuals in an observed community, then comparing that observed distance to that obtained for null communities generated from a random assemblage of taxa within communities, normalized by the standard deviation of phylogenetic distances in the null communities, that is, when species are randomized across the tips of the phylogeny (Kembel et al., 2015). In short, MPD provides a measure of the overall patterns of relatedness or similarities among members of a community, compared to that expected from a random assemblage of taxa within or among communities.

A mean MPD across all samples that is not significantly different than zero indicates random association of members of an assemblage, that is, no distinct pattern of genetic relatedness among members within a community. Significant deviations from mean MPD of zero indicate either of two things. First, a mean MPD that is significantly greater than zero is correlated with phylogenetic over‐dispersion, that is, broader representation of fungal lineages within a leaf community than expected at random where co‐occurring taxa are distantly related. Second, a mean MPD that is significantly less than zero suggests phylogenetic clustering where co‐occurring fungi within a community are composed of more closely related taxa than expected at random, that is, fungal communities are enriched for specific, closely related fungal lineages (Kembel et al., 2015).

### 2.8 Regional patterns of phylogenetic diversity across spatial scales

We investigated whether patterns of phylogenetic diversity increased with increasing spatial scales across all samples. We assessed the pattern of phylogenetic diversity expressed as MPD within an assemblage at four different spatial scales: within‐host (individual leaves), plot, block and site. For leaf, we calculated within‐community MPD at individual leaf—one leaf comprising one community (we collected one leaf per plant). For the plot, block and site levels, sample abundances were summed accordingly for each scale, for example, for the plot scale, all leaf microbiome samples belonging to one plot were aggregated by summing their OTU abundances and analyzing these summed abundances as a single community, that is, within‐plot level.

Mean phylogenetic distance was calculated using the standardized effect size (SES_mpd_), weighted by abundance, as implemented in the program picante (Kembel et al., 2015). We ran 999 randomizations with 1,000 iterations against the null model “taxa labels,” which shuffles tip labels among all taxa within that particular community, thus generating the random assemblages of taxa (the null communities) using the picante in R v3.2 (R Development Core Team, 2016). The significant difference between MPD and the null expectation of zero was tested using a two‐tailed *t* test at the 95% confidence level while comparisons among MPD of each treatment at each scale relative to control were performed using an Analysis of Variance (ANOVA) test, followed by post hoc Tukey test for multiple comparisons.

As fertilization and herbivore exclusion can have differential impacts on these communities, we calculated MPD across spatial scales for each of the four treatments separately: fertilization (NPK), fertilization with herbivore exclusion (NPK_H‐), herbivore exclusion without fertilization (C_H‐) and unmanipulated control plots (C). This allowed for assessing the overall patterns of phylogenetic diversity across the Great Plains region with respect to fertilization and herbivore exclusion at different spatial scales.

### 2.9 Effects of fertilization and herbivory on phylogenetic diversity at local scales

Next, we examined how fertilization and herbivore exclusion treatments impact phylogenetic diversity patterns at local scale, that is, within each of the sites (Minnesota, Iowa, Kentucky and Kansas). Within each site, we calculated within‐community MPD at individual leaf—one leaf comprising one community—for each of the four treatments separately. We also conducted analyses of MPD for microbes summed at plot and block levels for each of these treatments in order to assess if the impacts of the treatments within each site can be influenced by the spatial scale at which the fungal communities are assessed. For the plot and block levels, samples were binned accordingly for each scale, for example, all leaf microbiome samples belonging to one plot were aggregated, and that plot serve as one “community.” Analyses and calculation of MPD were done similarly as described above.

### 2.10 Phylogenetic beta‐diversity

While fertilization and herbivory can impact phylogenetic diversity patterns among fungal communities, environmental differences can lead to changes in community composition among sites. This can, in turn, lead to shifts in phylogenetic composition among communities (phylogenetic beta‐diversity) due to the loss or gain of specific taxa. We quantified phylogenetic beta‐diversity among the four regional sites as well as within each of the site. We used the function comdist() from picante in R, wherein the intercommunity phylogenetic distance matrix was generated between pairs of fungal taxa drawn from two distinct communities based on the mean MPD (similar to Bray–Curtis distances), and weighted by abundance.

Using the intercommunity phylogenetic distance described above, we investigated the sources of variation in phylogenetic dissimilarity among communities in order to determine the best predictor of phylogenetic turnover (analogous to among‐community dissimilarity, beta‐diversity) by performing a Permutational Multivariate Analysis of Variance (PERMANOVA). We tested for the effects of site, nutrient addition (fertilization) and herbivore exclusion (fence) using the model: *Y* ~ Site/Block/Plot/Plant + Site * Fertilization * Fence. The first term (Site/Block/Plot/Plant) accounts for the nested structure of the experimental set‐up and the second accounts for the main effects of site and treatments and their interactions; Y is the intercommunity phylogenetic distance. PERMANOVA analysis was done using the adonis() function in the vegan. We conducted 999 permutations, using site as a stratum in the permutations, which constrains permutations to samples within a site as we are interested in estimating OTU responses averaged across all sites. In order to examine whether shifts in phylogenetic distances were mirrored by changes at higher taxonomic levels (e.g., phylum, class, etc.), we summed the sequence abundance of each OTU—the ones included in ghost tree—at each of the seven taxonomic level and performed PERMANOVA analyses on the Bray–Curtis distances for each dataset representing diversity at different phylogenetic levels.

To further examine and visualize patterns of phylogenetic dissimilarity among communities, we used the intercommunity phylogenetic distance matrix to calculate the nonmetric multidimensional scaling (NDMS). We performed this analysis across our entire dataset, that is, across all sites and treatments.

### 2.11 Nonphylogenetic diversity metrics

In order to give better context to fungal phylogenetic diversity analyses, we calculated a metric of nonphylogenetic (i.e., species) diversity of microbes, Shannon's Diversity Index (Shannon, 1948), which accounts for the abundances, and OTU richness in vegan. We conducted similar diversity analyses mentioned above but using traditional species diversity metric. We also computed among‐community dissimilarity (equivalent to beta‐diversity), weighted by abundance, using Bray–Curtis method. Patterns were visualized on NMDS ordination. In these analyses, we only included OTUs that were incorporated in the phylogenetic diversity (*n* = 1,577), though these analyses were done at OTU level rather than at taxon level, that is, OTUs were not collapsed into consensus taxa as was done in the phylogenetic analyses. In addition, we performed a separate PERMANOVA analysis at different taxonomic levels, for example, phylum and class where OTUs were collapsed into each taxonomic level (Appendix S1).

## 3 RESULTS

### 3.1 Fungal OTU and taxa summary statistics

**Figure 2:**
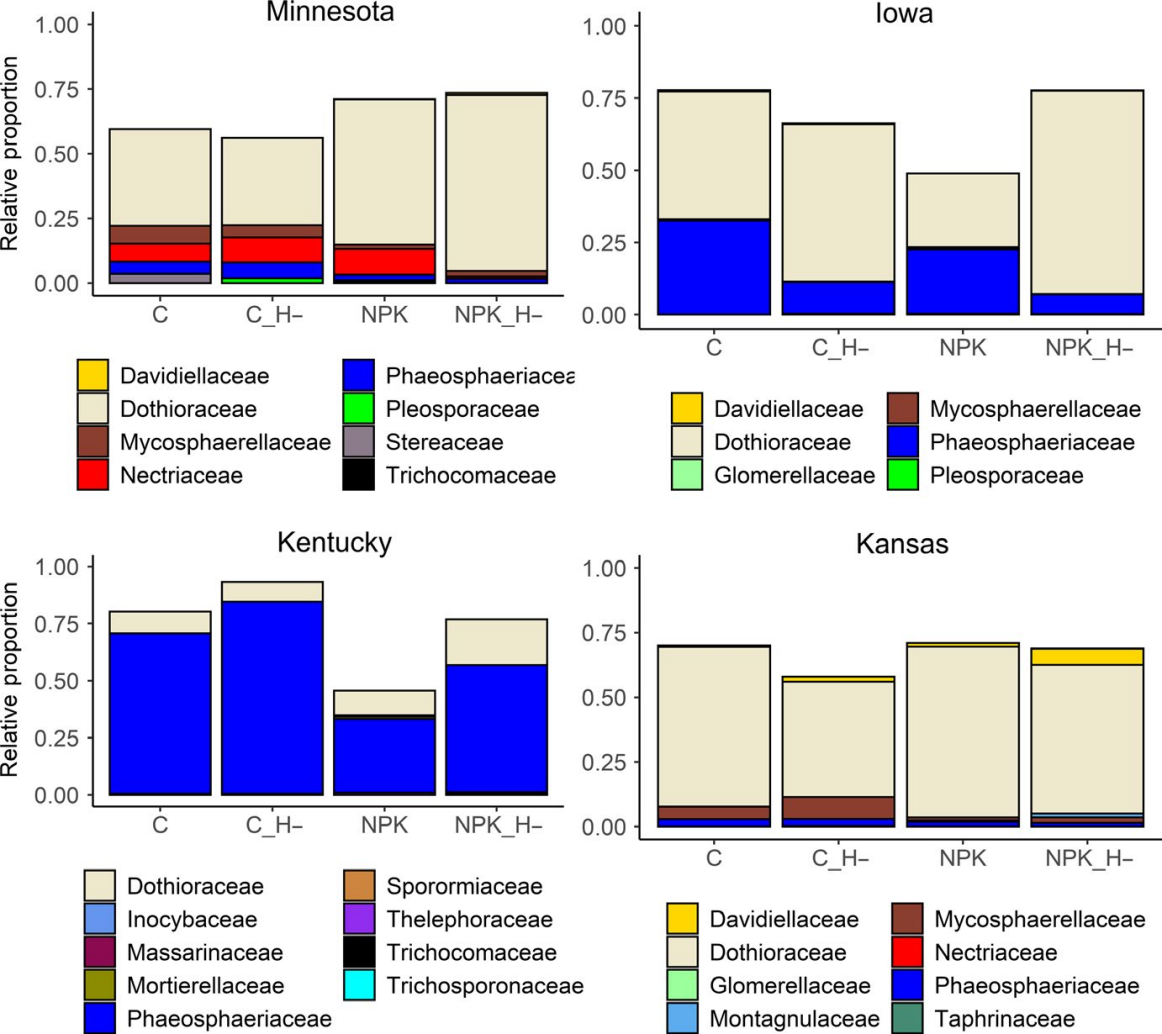
Most abundant fungal families for each treatment at each site. Values are based on proportion of raw sequences divided by all raw sequences within that particular treatment

Of the 176 *A. gerardii* leaf samples collected, genomic DNAs for seven samples generated no PCR amplicons, so 169 genomic DNA samples were sequenced. After quality filtering, a total of 14,261,237 sequences were obtained from these samples, and 2,772 OTUs were delineated at the 97% sequence identity level. Of these, 812 (30%) OTUs had no hits in the UNITE database. Those having a match in the database and that can be assigned at the phylum level represented six phyla. Ascomycota was the dominant phylum (92% of total assigned OTUs) across all treatments and sites, followed by Basidiomycota ([7%], Table S1). The remaining phyla (Glomeromycota, Rosellomycota and Chytridiomycota, in that order), as well as those belonging to “Zygomycota,” comprise about 1% of the total sequences. Unassigned OTUs (i.e., those 812 OTUs classified as fungi not but assigned to a phylum) comprised <1% of the total sequences. Members of those classified as “Zygomycota” were present primarily in Minnesota and Kentucky, but negligible in Kansas (sequence count = 33, <0.1%) and absent from all host leaves in Iowa (Table S1). The most dominant class across our dataset, Dothideomycetes, comprised ~84% of all assigned sequence reads, followed by Tremellomycetes (5.1%), Microbotryomycetes (1.9%) and Sordariomycetes ([1.5%], Table 1). The five most abundant fungal families varied among the four regional sites, although Dothioraceae and Phaeosphaeriaceae were the two most abundant families across sites (Figure 2).

**Table 1:** Relative percent abundance and raw sequence counts of OTUs assigned to top 10 most abundant classes across all samples

Class	Absolute sequence count	Relative % abundance
Dothideomycetes	8,345,544	89.81
Tremellomycetes	508,414	5.47
Microbotryomycetes	196,691	2.12
Sordariomycetes	150,110	1.62
Agaricomycetes	36,809	0.40
Cystobasidiomycetes	19,800	0.21
Eurotiomycetes	17,122	0.18
Leotiomycetes	6,566	0.07
Pezizomycetes	2,854	0.03
Taphrinomycetes	2,558	0.03

### 3.2 Regional patterns of phylogenetic diversity across spatial scales

**Figure 3:**
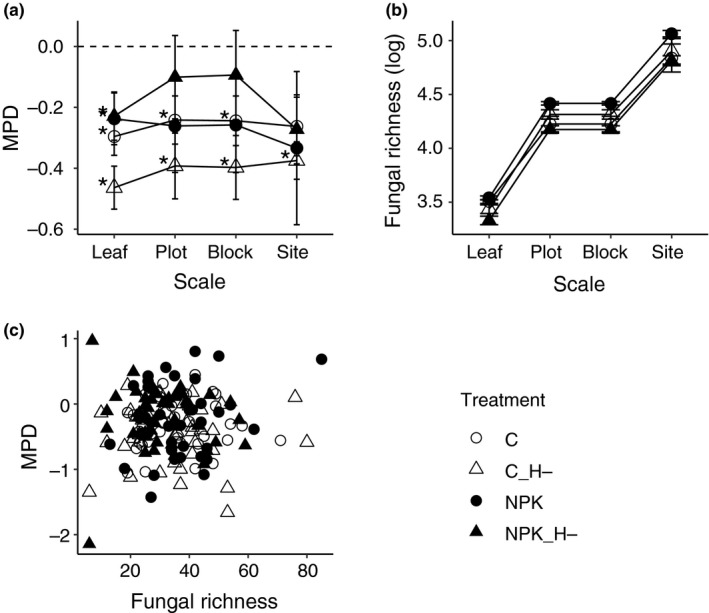
Cumulative (a) phylogenetic diversity (b) and fungal richness at different spatial scales. Each point is the mean MPD value or cumulative species richness summed to each scale; error bars are ± *SE*. For phylogenetic diversity, negative MPD values indicate phylogenetic clustering while positive mpd values indicate over‐dispersion. Asterisks (*) denotes mean MPD significantly different from zero, *p* < .05. Treatments: control (C), C_H‐ (herbivore exclusion without fertilization), NPK (fertilized) and NPK_H‐ (fertilization without herbivores). (c) MPD was not significantly correlated with fungal richness based on linear regression analysis

We first assessed the regional patterns of within‐community phylogenetic diversity across all samples. Phylogenetic relatedness (or similarity) expressed as mean MPD neither declined nor increased with increasing spatial scale, that is, the degree of phylogenetic relatedness remained constant from plot to site level (slope of the regression lines). Furthermore, phylogenetic relatedness remained the same across spatial scales in different experimentally imposed environment (i.e., control, fertilized, and fenced; Figure 3a). In contrast, both fungal OTU richness and Shannon diversity increased with increasing spatial scale (Figure 3b), although this increase was not associated with a larger representation of the phylogenetic tree. Moreover, the mean MPD was not significantly correlated with fungal richness or diversity across all leaves (Figure 3c). Thus, the increase in fungal richness or diversity were likely due to increase in fungal taxa that were drawn from similar phylogenetic clades (i.e., closely related taxa).

In addition, fungal communities were neither more closely related nor distantly related than expected across most spatial scales (i.e., plot to site level), as the mean MPD did not significantly deviate from zero (Figure 3a, Table 2). However, at the smallest scale (i.e., at within‐host/leaf scale), fungal endophytes within leaves across the region tended to be closely related, that is, phylogenetically clustered regardless of the environment (Figure 3a, Table 2). Thus, fungal phylogenetic diversity can be maintained across large spatial scales, except at the smallest, within‐host/leaf scale.

**Table 2:** Significance test of MPD treatment means at each scale across all samples from randomness (MPD = zero)

Treatment	Mean	*p*‐value
Leaf		
Control (C)	−0.296	**<.001**
Control‐Fenced (C_H‐)	−0.463	**<.001**
Fertilized (NPK)	−0.237	**.008**
Fertilized & Fenced (NPK_H‐)	−0.228	**.006**
Plot		
Control (C)	−0.241	**.012**
Control‐Fenced (C_H‐)	−0.392	**.004**
Fertilized (NPK)	−0.261	.118
Fertilized & Fenced (NPK_H‐)	−0.101	.477
Block		
Control (C)	−0.244	**.013**
Control‐Fenced (C_H‐)	−0.397	**.003**
Fertilized (NPK)	−0.257	.130
Fertilized & Fenced (NPK_H‐)	−0.094	.536
Site		
Control (C)	−0.263	.071
Control‐Fenced (C_H‐)	−0.375	**.009**
Fertilized (NPK)	−0.334	.276
Fertilized & Fenced (NPK_H‐)	−0.272	.095

Note

Significant factors are in bold text.

### 3.3 Effects of fertilization and herbivory on phylogenetic diversity at local scales

**Figure 4:**
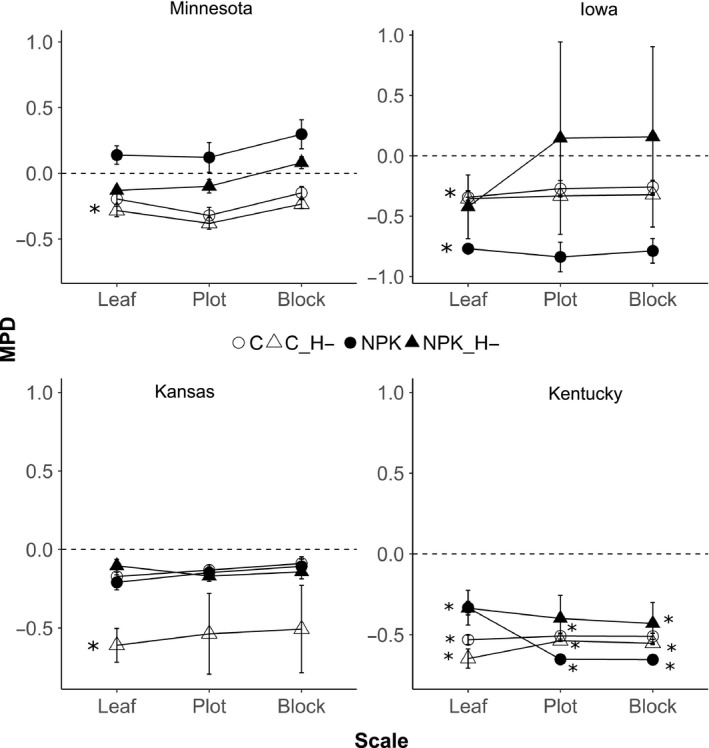
Cumulative phylogenetic diversity pattern within each site. Each point is the mean MPD value of abundances summed to each scale; error bars are ± *SE*. For phylogenetic diversity, negative MPD values indicate phylogenetic clustering while positive MPD values indicate over‐dispersion. Dashed line is zero; asterisks (*) denotes mean MPD significantly different from zero *p* < .05

The effects of fertilization and herbivory treatments on phylogenetic diversity of fungal endophyte assemblages at local scale (i.e., within each site) varied in magnitude and direction among the four regional sites (Figure 4). Fungal endophytes within leaves tended to be more closely related than expected under elevated nutrients in Iowa (mean NPK MPD = −0.769, *p* < .001, leaf scale) but not in the other three sites. In contrast, in the absence of large herbivores (C_H‐), fungal microbiomes within a host were comprised of phylogenetically closely related members than expected within all the four regional sites (Figure 4).

Fungal microbiomes within a leaf, plot and block under unmanipulated (control) as well as under fertilized without herbivore (NPK_H‐) environment showed random phylogenetic associations in Minnesota, Iowa and Kansas, but not in Kentucky. In Kentucky, fungal assemblages of plants under these environments showed significant phylogenetic clustering as members within a leaf, plot and block tend to be more closely related than expected (Figure 4). Thus, the effect of fertilization and herbivory treatments at local scales depended on site.

### 3.4 Phylogenetic beta‐diversity

**Figure 5:**
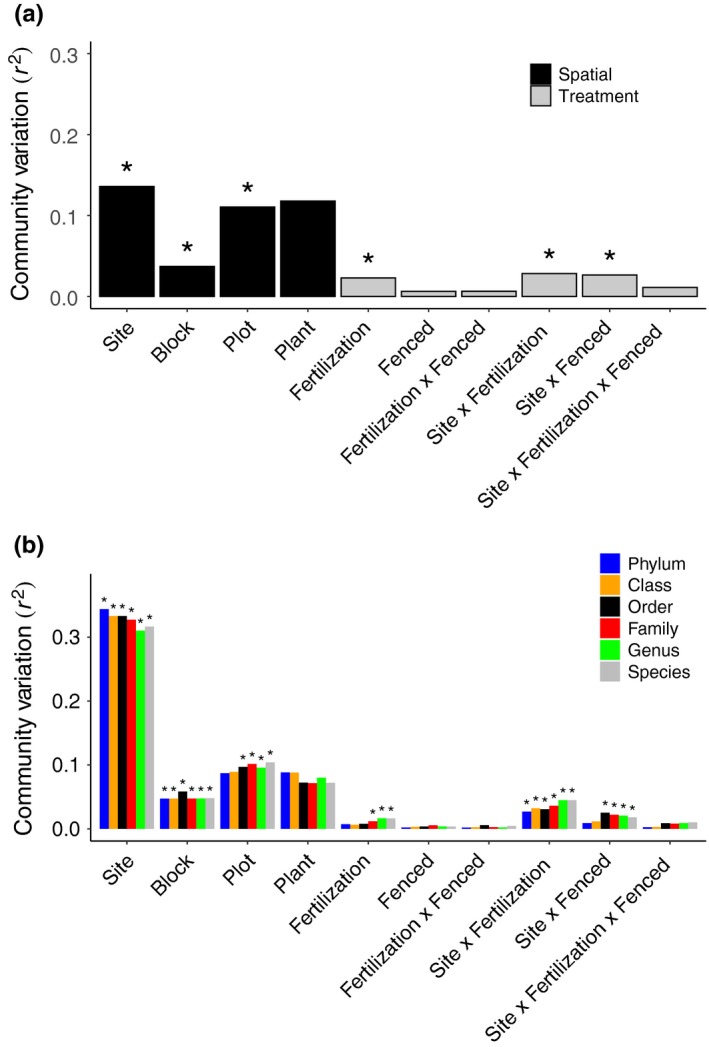
PERMANOVA analysis using (a) phylogenetic distances among fungal OTUs and (b) using Bray–Curtis distances at different taxonomic levels. Effects of site and treatments (fertilization and herbivore exclosure [fenced]), with *R* ^2^ value from PERMANOVA analysis plotted for factors to compare the relative effects of experimental factors on phylogenetic turnover and community compositional turnover among fungal endophyte communities. Asterisks (*) are *p* < .05. For (b), only OTUs included in the phylogenetic analyses were used in this analysis

Environmental differences among sites might lead to shifts in phylogenetic beta‐diversity, that is, phylogenetic dissimilarity among communities in different sites. The endophyte communities at each site tended to be drawn from distinct phylogenetic clades as members within each site were more closely related to one another than to endophytic communities from different sites (*R* ^2^ = .136, *p* = .002). This was further supported by the turnover in the most dominant groups at the family level across the four sites. Whereas *Phaeosphaeriaceae* was the most abundant family in Kentucky, and the second most abundant in Iowa, it was much less abundant at the other two sites (Figure 2). Similarly, *Steraceae* was abundant in Minnesota but largely absent at the other three sites. Phylogenetic composition of endophytes also varied among blocks (*R* ^2^ = .037, *p* = .005) and plots (*R* ^2^ = .110, *p* = .045) within all sites. In contrast, endophyte communities among leaves did not differ in composition as there was no shifts in phylogenetic composition among communities at smaller, individual leaf scale (*R* ^2^ = .118, *p* = .945, Figure 5a).

Fertilization treatment tended to select for fungal taxa that were drawn from distinct phylogenetic clades as there were significant shifts in phylogenetic compositions across all samples based on PERMANOVA analysis (*R* ^2^ = .022, *p* < .01). However, these shifts in taxa occurred only at lower taxonomic levels, from family down to species (Figure 5b). The effects of fertilization, however, were not consistent across sites, as indicated by significant interactions between site and nutrient addition (Figure 5). In contrast, herbivore exclusion had no significant influence on phylogenetic compositions among communities (*R* ^2^ = .006, *p* = .160).

**Figure 6:**
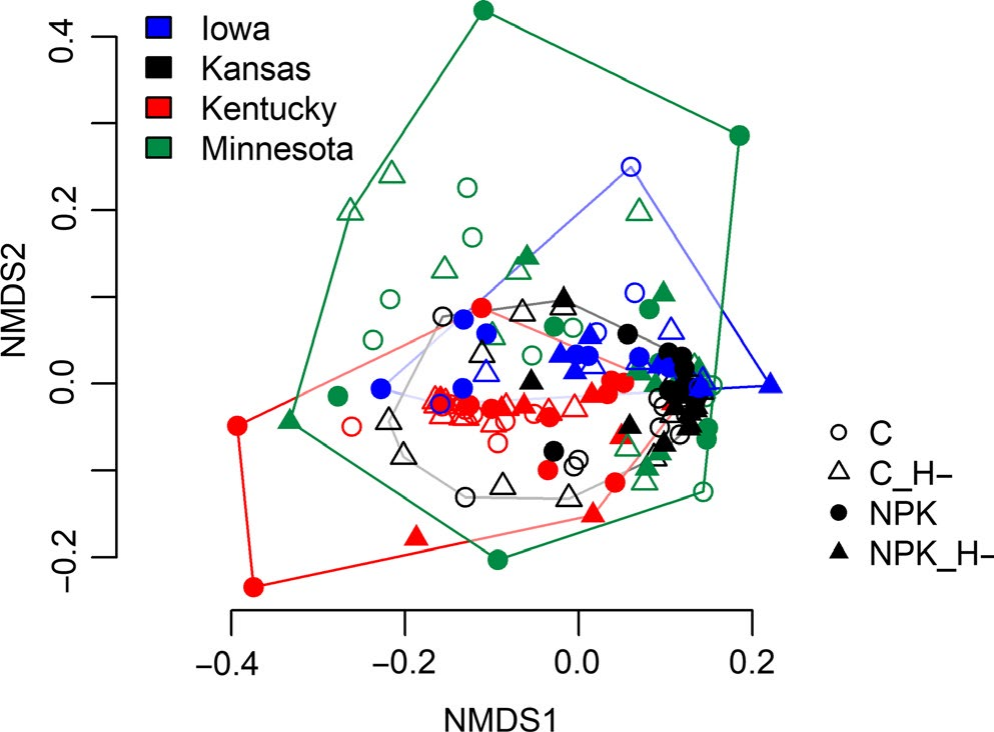
Nonmetric multidimensional scaling (NMDS) ordinations based on pairwise, abundance‐weighted mean phylogenetic distances across all samples. Each point represents a pair of leaves

The NMDS ordination plots illustrate the community variation underlying the PERMANOVA results. Phylogenetic dissimilarity of all leaf pairwise comparisons among the four treatments across all sites showed a weak but stronger clustering by site than by treatment (Figure 6). The NMDS ordination of intercommunity phylogenetic distances among taxa across all samples, a measure of the phylogenetic beta‐diversity, showed stronger differentiation among sites than treatments. This indicates that fungal taxa are more closely related within each site than between sites, and, although clades changed, treatments did little to modify the relatedness among taxa within each host. In addition, community turnover based on Bray–Curtis distances (Figure S3) showed stronger clustering by site than treatments, in parallel to phylogenetic turnover.

## 4 DISCUSSION

This study provides insights into the changes in phylogenetic relatedness of the plant fungal microbiome in response to pervasive environmental changes across spatial scales. We report two key findings. *First*, at large spatial scales, while fungal richness and diversity increased, foliar fungal communities of *A. gerardii* were comprised of taxa showing random phylogenetic associations regardless of the environment (i.e., control, fertilized or fenced) across the Great Plains region. However, there were substantial shifts in endophytic and phylogenetic composition among communities across the region, indicating that within each site (e.g., Minnesota, etc.) distinct but similarly diverse fungal communities are maintained. *Second*, within‐host/leaf scale fungal endophytes in different environment (i.e., treatments) across the region tended to be comprised by closely related taxa, though, we observed no phylogenetic turnover among communities. In addition, within each site, nutrient addition and herbivory have varying effects at different regional sites. These results suggest that the direction and magnitude of the outcomes of environmental modifications on fungal microbiomes likely depend on the spatial scale considered and can be constrained by site differences due to local environmental conditions influencing microbial diversity and composition.

### 4.1 Maintenance of diversity at large spatial scale

The degree of phylogenetic relatedness among co‐occurring members within a community depends on the relative strength of processes operating at each scale (Chave, Chust, & Thébaud, 2007; Mayfield & Levine, 2010; Morlon et al., 2011) and typically declines with increasing spatial scale (Cavender‐Bares et al., 2009; Harmon‐Threatt & Ackerly, 2013). Here, despite increasing fungal diversity and richness, we found that random phylogenetic associations, that is, no discernable phylogenetic structure among co‐occurring fungal taxa within a community (hence, phylogenetic diversity) was maintained across larger spatial scales (i.e., from plot to site level). This result suggests that across the Great Plains region, fungal communities harbored diverse group of fungi that are composed of a random mixture of closely and distantly related taxa. Under different treatments, these random phylogenetic associations also remained relatively unchanged across the region, suggesting that diversity might, in part, possibly have been maintained due to these fungal taxa being broad environmental generalists that are able to persist under different environment. For instance, we found fertilization with or without herbivores did little to modify the (within‐community) phylogenetic diversity of foliar fungal communities from plot to site scales.

While the endophytic communities at a site were random with respect to phylogeny, there was a high degree of compositional turnover in communities among the sites based on the phylogenetic structure among communities and mirrored by shifts in the abundant fungal OTUs (based on Bray–Curtis distance). These suggest that while diversity was maintained across the region, each site harbored phylogenetically distinct but similarly diverse pool of fungal communities, possibly due to dispersal limitation (Peay, Bruns, Kennedy, Bergemann, & Garbelotto, 2007; Talbot et al., 2014). Dispersal limitations at large spatial scale can constrain the regional pool of fungal communities available to colonize a given host. Distant sites were then likely drawing from different taxon pools causing substantial community and phylogenetic turnover among sites. For instance, at the family level, the dominant groups were variable across sites, with some families unique to one site (e.g., *Steraceae* in Minnesota) or two sites (*Plectosphaerellaceae* in Kansas and Iowa). Such differences have a significant impact on the degree of phylogenetic relatedness among members among sites. These results also imply that at larger spatial scales, for example, hundreds of kilometers among sites, dispersal limitation can be the predominant force in shaping foliar fungal communities of *A. gerardii*. Alternatively, it is also likely that these patterns might be due to other unmeasured environmental variables that are promoting growth of different fungal taxa at deeper clades as patterns arising from dispersal limitation can be confounded by site or local environmental differences. However, further studies incorporating additional sites (or testing for spatial autocorrelation) might be needed in order to make a strong inference or generalizations about what causes among site differences on these fungal communities (Seabloom et al., 2019).

It is, however, possible that these random phylogenetic associations we observed might be due to loss of taxonomic resolution arising from pooling different OTUs into the same taxon in the phylogenetic tree. If the evolution of the traits is occurring at the true species or population level, lack of taxonomic resolution at this level will result in random phylogenetic patterns. In addition, our results might not reflect the entire fungal communities as only a subset of taxa were included in the phylogenetic tree. For instance, had all taxa been included we might have observed different patterns, depending on the phylogenetic affinities of excluded taxa, for example, if more distantly related than closely related taxa were excluded. We note, however, that the nonphylogenetic diversity metric (i.e., OTU richness, Shannon diversity, and Bray–Curtis distances), diversity and compositional patterns were relatively similar when comparing between full OTU dataset and the subset OTUs (Figures S1 and S2, Table S2).

### 4.2 Effects of environmental modifications at local scale

The effects of elevated nutrients and herbivory are more pronounced at small scale, that is, leaf scale among and within each site. Phylogenetic clustering is often observed as a result of environmental filtering (Webb et al., 2002). Contrary to our expectations, here, we found phylogenetic clustering of co‐occurring members within a community across all environment (i.e., control, fertilized and fenced) at leaf scale when summed across the region. These results were in parallel with other studies where phylogenetic clustering was more apparent at smaller scales, (e.g., Parmentier et al., 2014; Ulrich et al., 2014).

Within each site, endophytes within a leaf also tend to be more closely related than expected by chance, especially in the absence of herbivores. In the absence of herbivory, fungal assemblages exhibited increased species diversity (Shannon diversity and richness), but this increase corresponded with selection for closely related species, and thus reduced phylogenetic diversity. In contrast, the effects of nutrient addition were only pronounced in two sites (Iowa and Kansas). A study of soil microbial communities within the experimental plots sampled in the current study found that nutrient addition had distinct effects on different phylogenetic groups of species (Leff et al., 2015), and our current results suggest that this effect on endophytic fungi might depend on local site. This stands in contrast to the effects of nutrients on the host plant community diversity: elevated nutrient supply can cause local plant species extinctions (Borer, Seabloom, Mitchell, & Cronin, 2014; Harpole et al., 2016) as well as reduction in plant phylogenetic diversity (Roth, Kohli, Rihm, Amrhein, & Achermann, 2015).

The different magnitude of herbivory and nutrient effects on species diversity (based on OTUs) and phylogenetic diversity among sites is likely due to differences in site's local biotic and abiotic environments. The different evolutionary lineages of fungal endophytes present at different sites are likely to have led to site‐dependent responses to herbivory and fertilization by the endophyte fungal communities at local scales. Endophyte fungal taxa are not well‐mixed across sites, and local communities drawn from these distinct regional microbial pools differ in their responses to important anthropogenic environmental changes.

These site‐dependent effects of nutrient addition and herbivory on phylogenetic diversity might, however, also arise if excluded taxa (in the phylogenetic tree) differ over the experimental variables and sites. That said, if there is a lot of functional redundancy among taxa, or function varies within genera and species, similar patterns will be observed.

## 5 CONCLUSION

The current experimental design, focused on the microbiome of a single host species, was replicated across sites in four US states, allowing us to separate the effects of environmental variation (nutrient addition and herbivore exclusion) from site environment and history. This design contrasts with many observational microbiome studies which have primarily been performed at single sites or across observed gradients. Our current multi‐site experimental design demonstrated that while fungal diversity increases, cumulative phylogenetic diversity remains relatively invariant across a wide range of spatial scales in spite of dispersal limitation that leads to turnover of clades among sites. In addition, local environmental filtering can reduce the phylogenetic diversity of the fungal endophytic microbiome, depending on the local site's biotic and abiotic conditions. While there are limitations to the phylogenetic analyses of fungal communities, this work can potentially provide new insights and directions linking species diversity with evolutionary history in assessing the impacts of pervasive environmental changes on endophytic fungal communities. This work adds to the growing body of work suggesting that environmental change can alter phylogenetic diversity, leading to domination by taxa within a few clades (e.g., Barnard et al., 2013; Placella et al., 2012), but extends it to demonstrate that the magnitude of this response by endophytic fungi is contingent on the clades present in the local community.

## CONFLICT OF INTEREST

We declare no competing interests.

## AUTHORS CONTRIBUTION

ETB, GM, LK, and EWS conceived idea, CYL and BC collected data and performed bioinformatics, CYL analyzed data and led writing of manuscript. All authors contributed significantly to revision and writing of the manuscript and gave approval for publication.

## Data Availability

Illumina sequences are available in NCBI SRA (BioProject ID: PRJNA564812).
