# Supplementary material for: Site‐specific responses of foliar fungal microbiomes to nutrient addition and herbivory at different spatial scales
Source: Ecol Evol. 2019 Oct 19;9(21):12231–44. doi: 10.1002/ece3.5711 (PMC6854330; doi:10.1002/ece3.5711)

Appendix S1

**Site-specific responses of foliar fungal microbiomes to nutrient addition and herbivory at different spatial scales**

Lumibao C.Y.^1^, Borer E.T.^1^, Condon B^1^, Kinkel L^2^, May G^1^, and Seabloom E.W.^1^

^1^ Department of Ecology, Evolution and Behavior, University of Minnesota, St. Paul, Minnesota 55108

^2^ Department of Plant Pathology, University of Minnesota, St. Paul, Minnesota 55108

*Phylogenetic tree construction (ghost-tree method)*

In the *ghost-tree* method, a “foundation” phylogeny is first built based on the 18s SSU rRNA, obtained from aligned databases of fungal 18S rRNA gene sequences in SILVA, with the Jukes-Cantor model of evolution as implemented in ghost-tree (Fouquier *et al*. 2016). The 18S rRNA gene region is evolutionary more conserved than the ITS region, allowing for alignment across divergent taxonomic groups. Next, sequences from ITS region were clustered into OTUs (the *Extension Sequence OTU*), and taxonomy were determined for each OTUs, and OTUs with the same consensus taxonomy were then grouped together. For each OTU group with same taxonomy, all sequences within that group were aligned, and a tree was built based on the alignment - called the *Extension trees*, thus generating multiple extension trees (from different aligned OTU groups) associated with consensus taxa (see Fig. 1 of Fouquier *et al*. 2016). The roots of these extension trees were then grafted onto the tip of the ‘foundation’ tree by taxa, creating the ‘ghost tree’. For further description see Fouquier *et al*. (2016). We used the computed ghost tree generated by the software that was built based on the UNITE v.7.2 database and publicly available in the Github repository. We used pre-build phylogenetic tree that was built using UNITE v.7.2 and SILVA v132 SSU. OTUs were re-clustered at 100 % identity threshold as recommended by the author.

*Taxonomic shifts among fungal communities*

We assessed the compositional differences between individual plants in the abundances of taxa representing different taxonomic levels for those OTUs included in the phylogenetic tree. This allows for evaluating whether compositional shifts based on OTUs above were mirrored by changes at specific taxonomic levels (e.g., phylum, class, etc.). Only OTUs that were included in the phylogenetic tree/analyses (n=1577, see Appendix S1) were included in these analyses. OTUs were collapsed into specific taxonomic levels, and sequence counts were summed to that level. We rarefied samples at n = 900 sequences and performed PERMANOVA analyses (described in the main text) at each level of taxonomic group.

**Table S1.** Raw sequence counts assigned to each phylum in different treatments across the four sites. C – control; C_H- herbivore exclosure; NPK – nutrient addition; NPK_H- nutrient addition with herbivore exclosure.

| **Site** | **Treatment** | **Ascomycota** | **Basidiomycota** | **Chytridiomycota** | **Glomeromycota** | **Rozellomycota** | **Zygomycota^*^** |
| --- | --- | --- | --- | --- | --- | --- | --- |
| Minnesota | C | 147054 | 16318 | 0 | 106 | 11 | 415 |
| Minnesota | C_H- | 322389 | 43847 | 150 | 54 | 0 | 271 |
| Minnesota | NPK | 256996 | 8824 | 0 | 115 | 43 | 612 |
| Minnesota | NPK_H- | 350322 | 35874 | 48 | 0 | 0 | 136 |
| Iowa | C | 502823 | 3449 | 0 | 0 | 0 | 0 |
| Iowa | C_H- | 728924 | 1924 | 6 | 0 | 0 | 0 |
| Iowa | NPK | 771524 | 8983 | 0 | 0 | 0 | 0 |
| Iowa | NPK_H- | 954525 | 3787 | 0 | 0 | 0 | 0 |
| Kentucky | C | 334028 | 6386 | 0 | 180 | 183 | 343 |
| Kentucky | C_H- | 499399 | 7568 | 0 | 41 | 0 | 129 |
| Kentucky | NPK | 288536 | 12549 | 0 | 244 | 83 | 3240 |
| Kentucky | NPK_H- | 176051 | 3823 | 0 | 17 | 0 | 178 |
| Kansas | C | 867939 | 185194 | 0 | 3 | 0 | 24 |
| Kansas | C_H- | 557434 | 141329 | 0 | 0 | 0 | 0 |
| Kansas | NPK | 1173952 | 119560 | 0 | 2 | 0 | 7 |
| Kansas | NPK_H- | 1115280 | 167020 | 0 | 2 | 0 | 2 |

- This classification was no longer a valid taxonomic rank

**Table S2.** PERMANOVA results using Bray-Curtis distances for the full dataset and subset data.

| *Full Dataset* |  |  |  |  |  |  |
| --- | --- | --- | --- | --- | --- | --- |
| **Factors** | **Df** | **SumsOfSqs** | **MeanSqs** | **F.Model** | **R2** | **p-value** |
| **Site** | 3 | 16.755 | 5.585 | 37.443 | 0.329 | **0.001** |
| **Block** | 4 | 2.940 | 0.735 | 4.928 | 0.058 | **0.001** |
| **Plot** | 17 | 7.929 | 0.466 | 3.127 | 0.156 | **0.001** |
| Plant | 49 | 3.135 | 0.131 | 0.876 | 0.062 | 0.951 |
| **Fertilization** | 1 | 0.770 | 0.770 | 5.164 | 0.015 | **0.002** |
| Fenced | 1 | 0.172 | 0.172 | 1.151 | 0.003 | 0.297 |
| Fertilization x Fenced | 1 | 0.290 | 0.290 | 1.946 | 0.006 | 0.060 |
| **Site x Fertilization** | 3 | 2.060 | 0.687 | 4.602 | 0.040 | **0.001** |
| Site x Fenced | 3 | 0.752 | 0.251 | 1.681 | 0.015 | 0.068 |
| **Site x Fertilization x Fenced** | 2 | 0.582 | 0.291 | 1.949 | 0.011 | **0.018** |
| Residuals | 104 | 15.513 | 0.149 |  | 0.305 |  |
|  |  |  |  |  |  |  |
| *Subset Data* |  |  |  |  |  |  |
|  | Df | SumsOfSqs | MeanSqs | F.Model | R2 | Pr(>F) |
| **Site** | 3 | 13.599 | 4.533 | 22.066 | 0.256 | **0.001** |
| **Block** | 4 | 2.056 | 0.514 | 2.502 | 0.039 | **0.002** |
| **Plot** | 17 | 6.512 | 0.383 | 1.865 | 0.122 | **0.001** |
| Plant | 24 | 4.327 | 0.180 | 0.878 | 0.081 | 0.988 |
| **Fertilization** | 1 | 1.034 | 1.034 | 5.033 | 0.019 | **0.001** |
| Fenced | 1 | 0.197 | 0.197 | 0.957 | 0.004 | 0.498 |
| Fertilization x Fenced | 1 | 0.258 | 0.258 | 1.254 | 0.005 | 0.240 |
| **Site x Fertilization** | 3 | 2.624 | 0.875 | 4.258 | 0.049 | **0.001** |
| **Site x Fenced** | 3 | 1.021 | 0.340 | 1.657 | 0.019 | **0.042** |
| **Site x Fertilization x Fenced** | 2 | 0.811 | 0.406 | 1.974 | 0.015 | **0.013** |
| Residuals | 101 | 20.748 | 0.205 |  | 0.390 |  |

**Figure S1**. Strong significant correlation of fungal richness within a community between full OTU dataset and subset OTU dataset at each spatial scale. For plot to site scale, OTU abundances were summed up to each corresponding category. Symbols are similar to Figure 3 of main text.


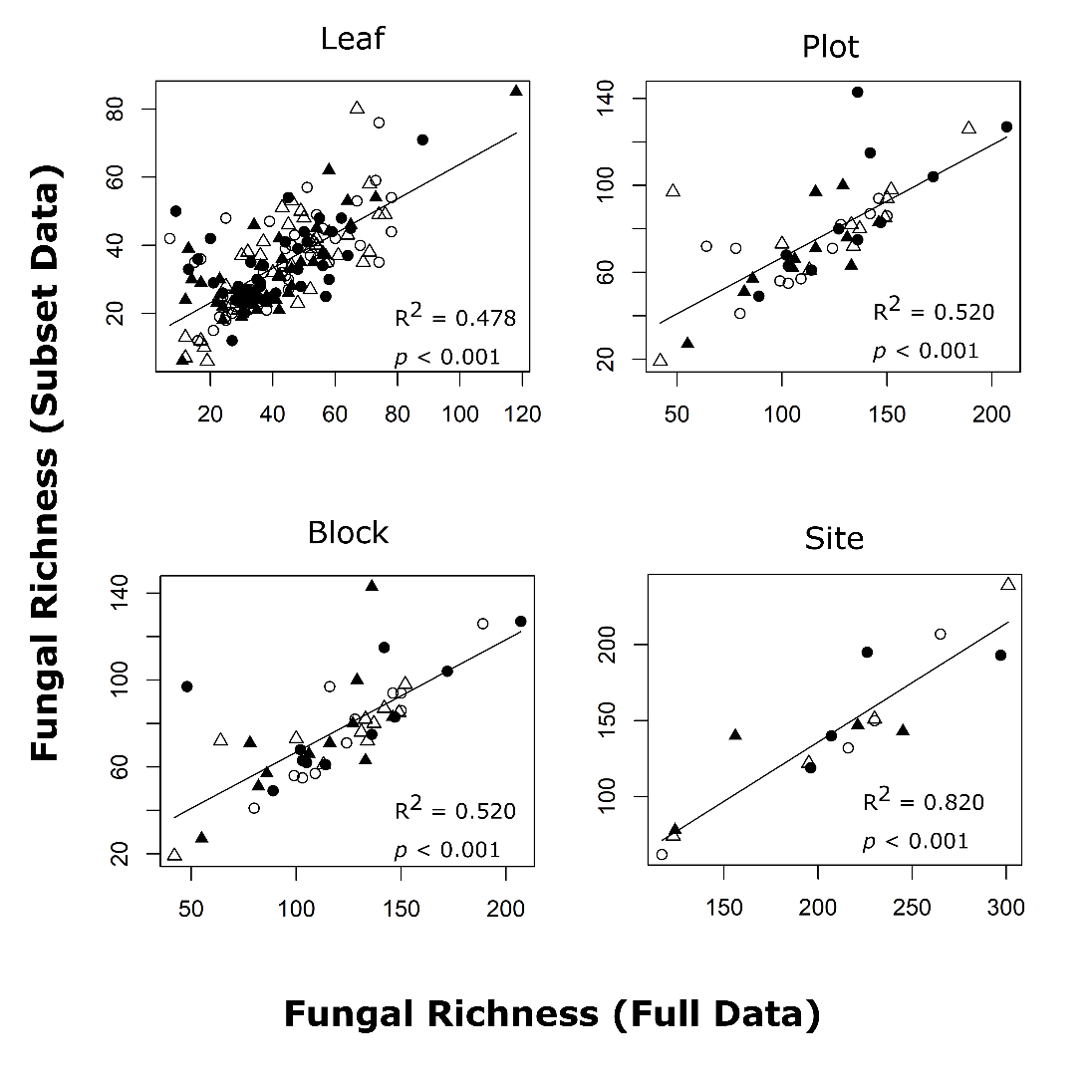


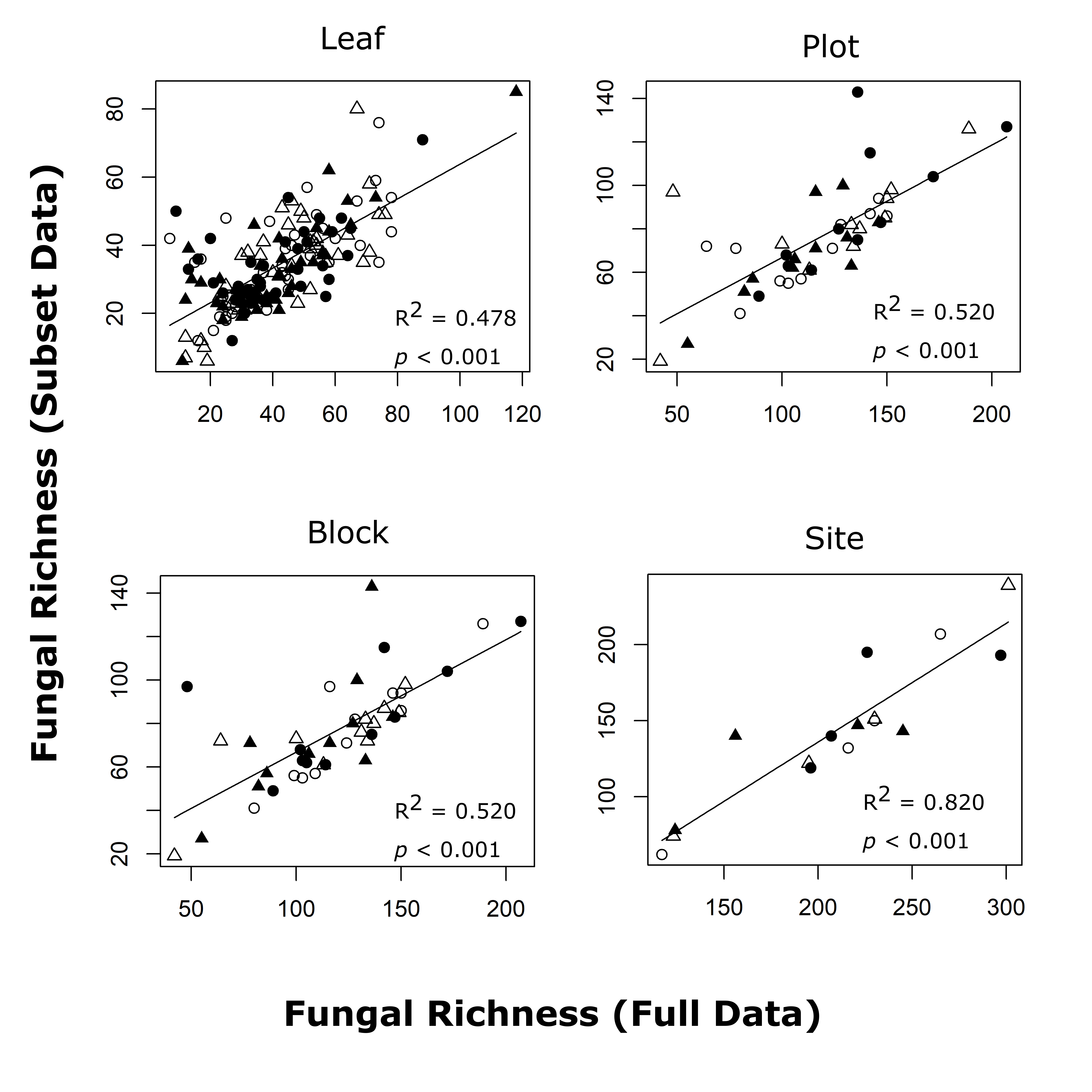


**Figure S2.** Strong significant correlation of Shannon diversity within a community between full OTU dataset and subset OTU dataset at each spatial scale. For plot to site scale, OTU abundances were summed up to each corresponding category. Symbols are similar to Figure 3.


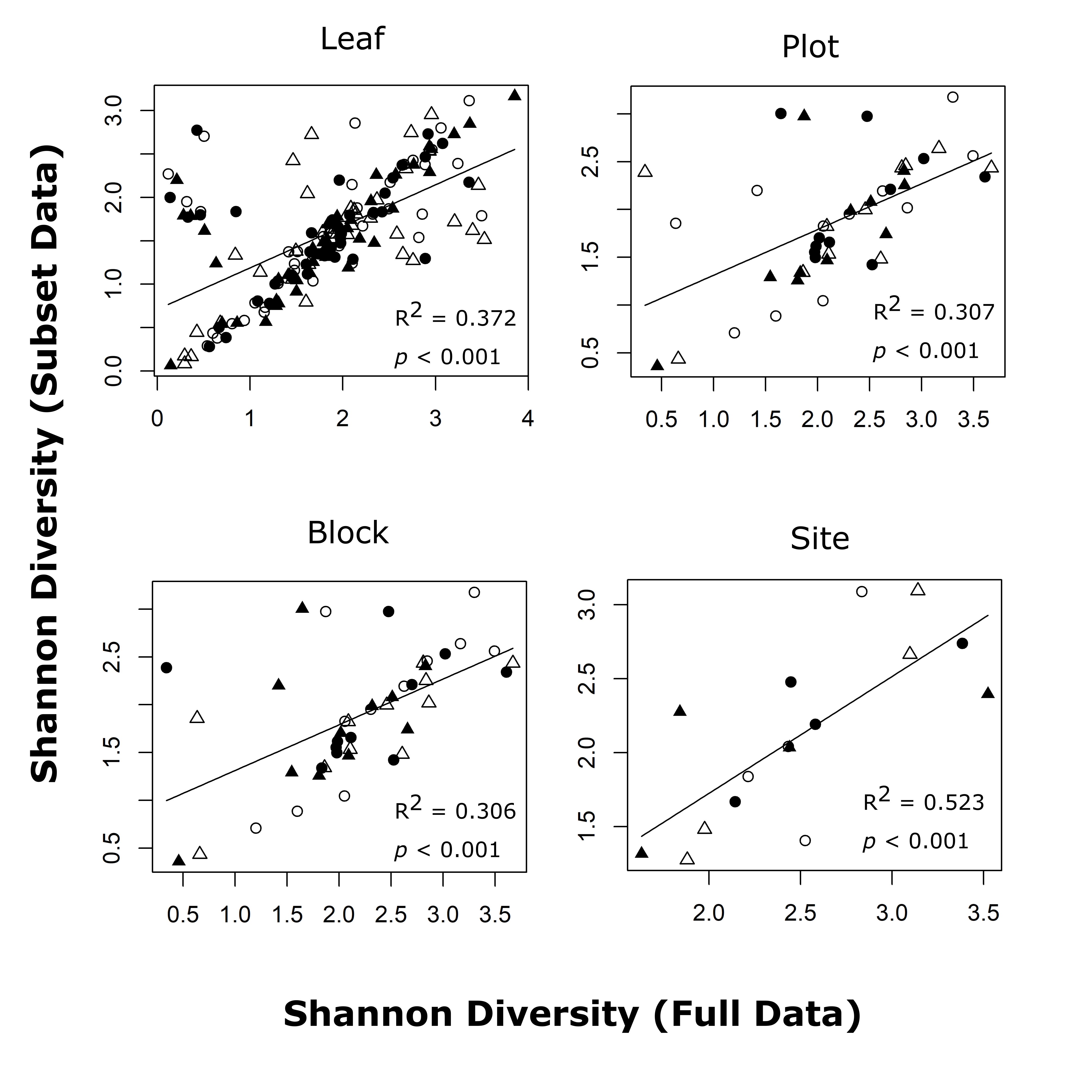


**Figure S3.** Non-metric multidimensional scaling (NMDS) ordinations based on pairwise, abundance-weighted Bray-Curtis distances across all samples. Ordihulls were drawn based on site clustering.


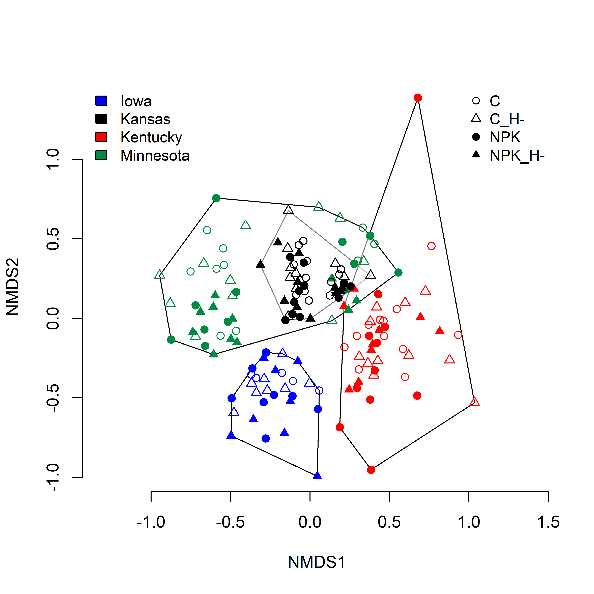

Supplement: Supplementary file 1 [file ECE3-9-12231-s001.docx]
